# Early adversity disrupts the adult use of aversive prediction errors to reduce fear in uncertainty

**DOI:** 10.3389/fnbeh.2015.00227

**Published:** 2015-08-27

**Authors:** Kristina M. Wright, Alyssa DiLeo, Michael A. McDannald

**Affiliations:** McDannald Lab, Department of Psychology, Boston CollegeChestnut Hill, MA, USA

**Keywords:** fear, prediction error, adolescent, adversity, Pavlovian, stress, PTSD

## Abstract

Early life adversity increases anxiety in adult rodents and primates, and increases the risk for developing post-traumatic disorder (PTSD) in humans. We hypothesized that early adversity impairs the use of learning signals -negative, aversive prediction errors–to reduce fear in uncertainty. To test this hypothesis, we gave adolescent rats a battery of adverse experiences then assessed adult performance in probabilistic Pavlovian fear conditioning and fear extinction. Rats were confronted with three cues associated with different probabilities of foot shock: one cue never predicted shock, another cue predicted shock with uncertainty, and a final cue always predicted shock. Control rats initially acquired fear to all cues, but rapidly reduced fear to the non-predictive and uncertain cues. Early adversity rats were slower to reduce fear to the non-predictive cue and never fully reduced fear to the uncertain cue. In extinction, all cues were presented in the absence of shock. Fear to the uncertain cue in discrimination, but not early adversity itself, predicted the reduction of fear in extinction. These results demonstrate early adversity impairs the use of negative aversive prediction errors to reduce fear, especially in situations of uncertainty.

## Introduction

Early adversity increases anxiety in adult rodents (Avital and Richter-Levin, 2005; Pohl et al., 2007; Tsoory et al., 2007; Franklin et al., 2010) and primates (Sánchez et al., 2005; Sanchez, 2006), and also increases the risk for post-traumatic stress disorder (PTSD) in humans (Kessler et al., 1997; Breslau et al., 1999; Cloitre et al., 2009; Xie et al., 2010). Risk for acquiring PTSD increases as more types of adversity are experienced (Breslau et al., 1999; Xie et al., 2012). Long-studied in the context of reward (Schultz et al., 1997; Fiorillo et al., 2003, 2008; Roesch et al., 2007; Niv and Schoenbaum, 2008), recent work highlights the role of prediction errors in aversive learning (McNally and Cole, 2006; McNally et al., 2011; Berg et al., 2014; Li and McNally, 2014; McHugh et al., 2014; Roy et al., 2014; Yau and McNally, 2015). Increased anxiety in adverse-experienced individuals, such as those with PTSD, may partially result from an inability to effectively utilize aversive prediction errors (APEs) to reduce fear. APEs are learning signals generated by a discrepancy between actual and predicted aversive outcomes and come in two varieties: “positive” and “negative”. “Positive” and “negative” do not refer to the value of the outcome, but rather to the direction of the discrepancy. Probabilistic reinforcement in Pavlovian fear conditioning is a straightforward setting in which APEs contribute to fear reduction.

During probabilistic reinforcement, a single cue predicts aversive foot shock on 25% of trials so the outcome of any particular trial is uncertain. Because this cue is initially neutral, there is no prediction the shock will occur. The first time the cue is followed by a shock, assigned an arbitrary value of 1.0, the result is surprising and aversive. The discrepancy between the actual shock and the prediction (1.0 − 0.0 = +1.0) results in a *positive* APE (+APE). The +APE is then broadcast to relevant brain regions to *strengthen* the cue-shock association and *increase* fear to the cue. When the same cue is encountered again, the prediction will be higher (0.5). If no shock occurs on this trial, the result is surprisingly better than expected. As a result there will be a negative discrepancy between the actual shock and the prediction (0.0 − 0.5 = −0.5). The result is a *negative* APE (−APE) that will *weaken* the cue-shock association and *reduce* fear to the cue. The use of APEs allows subjects to appropriately adjust levels of fear to cues that predict aversive events with uncertainty.

We hypothesize that early adversity disrupts the use of −APEs to reduce fear in uncertainty. To test this hypothesis, we gave adolescent rats a 10-day battery of adverse experiences. In adulthood, we assessed their ability to reduce fear via −APEs using a Pavlovian fear conditioning procedure. Three initially neutral cues were then associated with different probabilities of shock: 100, 25, and 0%. We have found that rats initially acquire high levels of fear to all cues, but come to reduce their fear to the 25 and 0% cues (Berg et al., 2014). Although −APEs could contribute to the reduction of fear to the 0% cue, this should dissipate over the course of discrimination because rats can reduce fear to this cue purely by discriminating the sensory features of the 100 and 0% cues. In contrast, reduction of fear to the uncertain, 25% cue requires the use of −APEs throughout discrimination because shock occurrence is unpredictable on a trial by trial basis. Finally, we assessed fear extinction to the 100% cue, another setting in which −APEs could contribute to the reduction of fear. We have previously found that rats using −APEs most effectively—achieving the lowest levels of fear to the uncertain cue during discrimination—most rapidly reduced fear during extinction (Berg et al., 2014). Here, we sought to directly test if Early Adversity had effects on extinction outside of the demonstrated ability to use −APEs to reduce fear during discrimination.

We found that in discrimination, Control rats came to show appropriate levels of fear to each cue: high to the 100%, little or no fear to the 0%, and modest fear to the uncertain cue. Early Adversity (EA) rats were slower to reduce their fear to the 0% cue and never fully reduced fear to the uncertain cue. The level of fear demonstrated to the uncertain cue during discrimination strongly and significantly predicted fear during extinction in all rats. Thus, increased adult anxiety, by way of Early Adversity, may specifically reflect an inability to use APEs to reduce fear. These results have implications for early adversity experiences and the long term, adult risk for anxiety disorders such as PTSD.

## Methods and materials

### Subjects

Subjects were 22 male Long Evans rats approximately 21 days old on arrival, obtained from Charles River Laboratories, and maintained on a 12-h light cycle (lights off at 6:00 PM). Rats were individually housed with food and water freely available. From arrival through postnatal day 56, rats were weighed three times per week: Monday, Wednesday, and Friday. Starting on P56, rats were maintained at 85% of their free-feeding body weight for the duration of Pavlovian fear conditioning. Water was always freely available. All protocols were approved by the Boston College Animal Care and Use Committee and all experiments were carried out in accordance with the NIH guidelines regarding the care and use of rats for experimental procedures.

### Apparatus

Forced swim took place in a clear, 10 L plastic cylinder. Tail pinch and cat hair exposure took place in a clear, plastic mouse cage free of bedding. Restraint occurred in a clear plastic restraint. The apparatus for Pavlovian fear conditioning consisted of eight individual chambers with aluminum front and back walls, clear acrylic sides and top, and a metal grid floor. Each grid floor bar was electrically connected to an aversive shock generator (Med Associates, St. Albans, VT). A single external food cup and central nose poke opening equipped with infrared photocells, were present on one wall. Auditory stimuli were presented through two speakers mounted on the ceiling of each chamber.

### Early adversity

**Figure 1:**
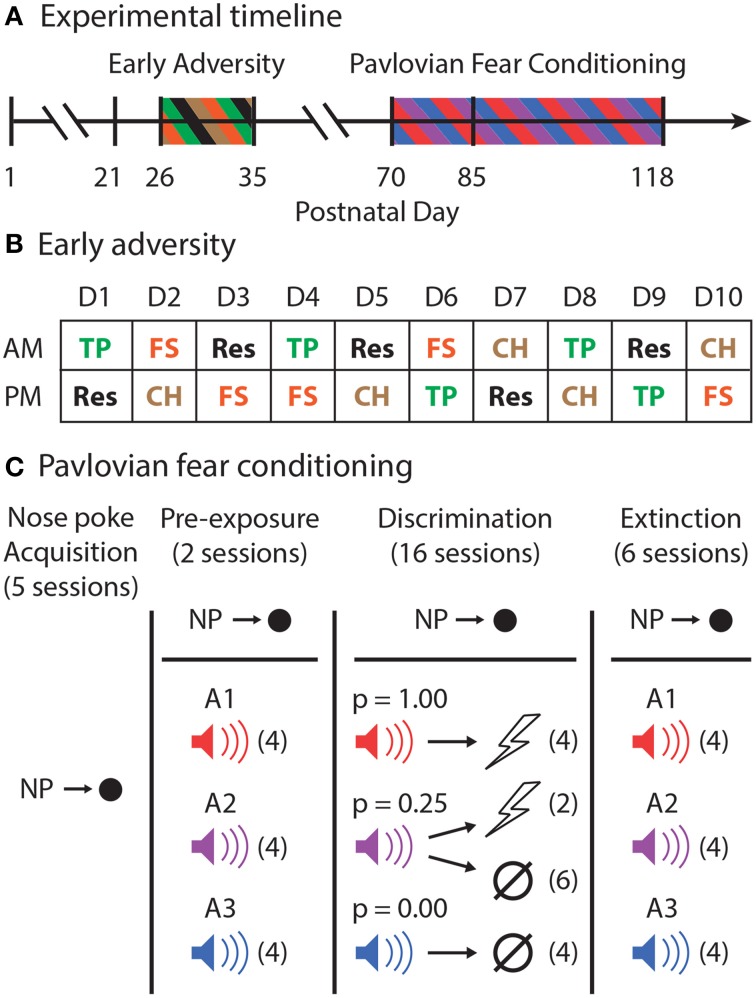
**Experimental outline. (A)** The experimental timeline is shown. Male, Long Evans rats arrived to the animal facility on postnatal day (P)21. From P26 through P35, half of the rats experienced Early Adversity. All rats were then allowed to mature to adulthood. Pavlovian fear conditioning began with nose poke shaping on P70. The first session of shock delivery during Pavlovian fear conditioning occurred on P85. **(B)** Early adversity consisted of morning (AM) and afternoon (PM) adverse experiences of one of four types: tail pinch (TP–Green), restraint (Res–Black), forced swim (FS–Orange), or cat hair exposure (CH–Brown). The exact order of these events for all rats over the 10 consecutive adverse experience days is shown. **(C)** In Pavlovian fear conditioning, rats first learned to nose poke to receive food pellets in five sessions. Rewarded nose poking was maintained for the entirety of Pavlovian fear conditioning. Rats were pre-exposed to all three to-be-conditioned cues, A1–red, A2–purple, and A3–blue, in two sessions receiving four cue presentations each session (# of trials in each session indicated by parentheses). During the 16 discrimination sessions, each cue was associated with a different probability of foot shock [A1, p(shock) = 1.00; A2, p(shock) = 0.25; and A3, p(shock) = 0.00]. A1 and A3 were each presented four times. A2 was followed by shock on two trials and was of no consequence on six trials. During six extinction sessions, all three cues were presented four times each in the absence of foot shock.

Rats arrived to the animal facility on postnatal (P) 21 (Figure 1A). From P26 to P35, all EA rats were exposed to a total of 20 adverse events. Each EA rat experienced four different adverse events five times: forced swim, tail pinch, cat hair exposure, and restraint. These adverse experiences were chosen because their stressful properties have been well-demonstrated (D'Angio et al., 1987; Melia et al., 1994; Kelly et al., 2011; Vazdarjanova et al., 2001). Each day, every EA rat received one adverse experience in the morning and a different adverse experience during an afternoon session, at least 4 h later. The order of adverse events (Figure 1B) was constructed so that no single day was the same as another.

#### Forced swim

EA Rats were placed in 10 L of 10°C water for 5 min. The water level was such that rats could neither reach the bottom nor the top of the container. When 5 min expired, the rat was dried vigorously with a towel and returned to his home cage.

#### Tail pinch

Tail pinch took place in a clear, plastic mouse cage free of bedding. A half-inch mini binder clip was placed on the base of each rat's tail for 5 min. When 5 min expired, the binder clip was removed and the rat was returned to his home cage.

#### Cat hair

A ball of hair obtained from a cat certified to be disease-free by a veterinarian was placed in a hair net and suspended from the wire ceiling of a clear, plastic mouse cage free of bedding. Each rat was placed in the cage containing cat hair for 5 min, and was returned to his home cage immediately following the exposure.

#### Restraint

Each rat was placed into a plastic restraint (Flat bottom restrainers, Braintree Scientific) for 30 min. When 30 min expired, the rat was promptly removed from the restraint, and returned to his home cage.

Half of the rats (EA; *n* = 11) were randomly selected to undergo the early adverse procedure. The remaining rats (Control; *n* = 11) arrived at the animal facility on the same day and were handled and weighed as EA rats, but they did not receive any adverse experiences. During the procedure, EA and Control rats were housed in separate rooms. Two weeks following the end of the adverse experience procedure, EA rats joined the colony room in which the Control rats were housed.

### Pavlovian fear conditioning

#### Nose poke acquisition

The design of the Pavlovian fear conditioning procedure is shown in Figure 1C. Prior to any behavioral sessions, rats were food-deprived to 85% of their free-feeding body weight and were fed specifically to maintain this weight through the behavioral procedure. Starting on P70, rats were shaped to nose poke for pellet delivery in the experimental chamber using a fixed ratio schedule in which one nose poke yielded one pellet. Shaping sessions lasted 30 min or until approximately 50 nose pokes were completed. Over the next 3 days, rats were placed on 3 days of variable interval (VI) schedules in which nose pokes were reinforced on average every 30 s (day 1), or 60 s (days 2 and 3). For the remainder of behavioral testing, nose pokes were reinforced on a VI-60 schedule independent of all Pavlovian contingencies.

#### Pre-exposure

The experimental design was completely within subjects. In two separate sessions, each rat was pre-exposed to the three auditory (A1-3) cues to be used in Pavlovian discrimination. These 42-min sessions consisted of four presentations of each cue (12 total presentations) with a mean inter-trial interval (ITI) of 3.5 min. The order of trial type presentation was randomly determined by the behavioral program, and differed for each rat during each session.

#### Discrimination

Starting on P85, and for the next sixteen sessions, each rat underwent Pavlovian discrimination training. Each of the 16, 54-min sessions consisted of 16 trials, with a mean ITI of 3.5 min. Each auditory cue (A1-3) was 10-s in duration, and was associated with a different probability of foot shock (0.5 mA, 0.5 s). Cue A1 always predicted shock [p(shock) = 1.00], cue A2 probabilistically predicted shock [p(shock) = 0.25], and cue A3 never predicted shock [p(shock) = 0.00]. For reinforced trials, the foot shock was administered 1 s following the termination of the auditory cue. A single session consisted of four A1 trials, six A2 no shock trials, two A2 shock trials, and four A3 trials. The order of trial type presentation was randomly determined by the behavioral program, and differed for each rat during each session.

#### Extinction

Six extinction sessions were given, one session per day, following the final discrimination session. The composition of these sessions was exactly like those for pre-exposure. Each 42-min session consisted of four presentations of each cue (12 total presentations) with a mean inter-trial interval (ITI) of 3.5 min. The order of trial type presentation was randomly determined by the behavioral program, and differed for each rat during each session.

### Statistical analyses

Body weights were analyzed for group differences using analysis of variance (ANOVA). Behavioral data from Pavlovian fear conditioning were acquired using Med Associates Med-PC IV software. The time stamp for every nose poke and event onset (cues and shocks) during each session was recorded automatically. Raw data were processed in Matlab to extract nose poke rates during two periods: the baseline, which was 20 s prior to cue onset, and the entire 10 s cue. A suppression ratio was calculated as follows: (baseline − cue)/(baseline + cue). A ratio of “1” indicated complete suppression of nose poking during the cue relative to baseline, indicative of a high level of fear. A ratio of “0” indicated no suppression of nose poking, indicating little or no fear (Pickens et al., 2009). A ratio < 0 indicates subjects increased their nose poke rate during the cue, relative to baseline. Suppression ratios were then analyzed with Statistica using repeated measures analysis of variance (ANOVA) and analysis of covariance (ANCOVA) with body weight as a covariate. Planned comparisons were performed using contrasts, and *post-hoc* comparisons were made with two-tailed *t*-tests.

A sliding-window analysis was performed to identify differences in suppression ratios to each of the three cues by each Control and EA rat throughout the two pre-exposure, and sixteen discrimination sessions. Starting with the first pre-exposure session, and for each cue separately, we took the mean suppression ratio from trial n → *n* + 3. For each window, we calculated a difference score (EA–Control) and also performed a one-tailed, independent sample *t*-test because we were testing a directional hypothesis. The sliding window was moved ahead one session at a time until all 15 windows had been analyzed (p1 → d2, p2 → d3, …d13 → d16). A significance level of *p* < 0.05 was used throughout.

## Results

### Body weight

All rats had free access to water and food from the day they arrived until they began the Pavlovian fear conditioning procedure. While Control and EA rats arrived at the same weight, EA rats gained weight more slowly than Controls following the early adversity procedure. In adulthood, Control and EA rats did not differ significantly in weight. These descriptions are supported by ANOVA [within factor: day (16); between factor: group (Control vs. EA)] for body weight that found a significant effect of day [*F*_(14, 280)_ = 1880.1, *p* < 0.01] and the day × group interaction [*F*_(14, 280)_ = 3.78, *p* < 0.01], but no main effect of group [*F*_(1, 20)_ = 2.01, *p* = 0.17].

### Baseline nose poke rates

During Pavlovian fear conditioning, Control and EA rats showed equivalent levels of baseline nose poking throughout pre-exposure, discrimination, and extinction. ANOVA [within factor: session (24); between factor: group (Control vs. EA)] for baseline nose poke rates revealed a significant effect of session [*F*_(23, 460)_ = 20.55, *p* < 0.01], but no effect of group or session × group interaction (Fs < 1, ps > 0.4). By the final extinction session, mean ± SEM baseline nose poke rates for each group were Control: 53.1 ± 9.9, and EA: 52.5 ± 6.0.

### Discrimination

**Figure 2:**
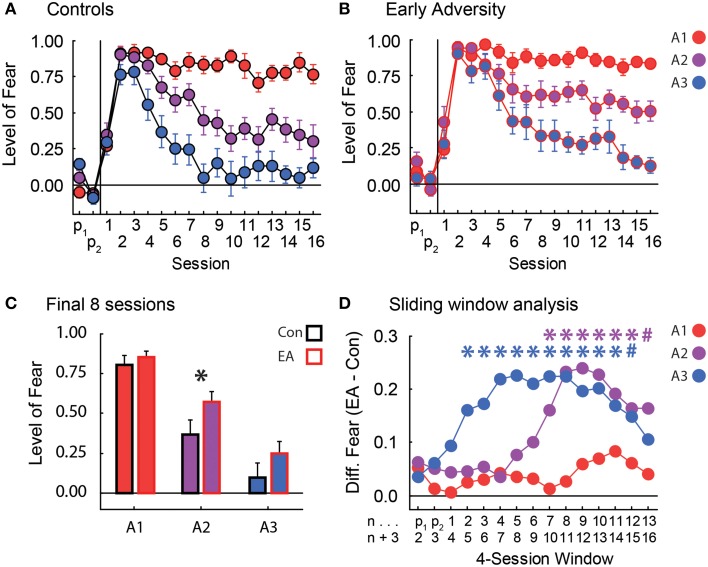
**Fear in discrimination. (A)** The level of fear (y-axis, suppression ratio) to the three cues [p(shock) = 1.00, red; p(shock) = 0.25, purple; and p(shock) = 0.00, blue] over the two pre-exposure sessions and the 16 Pavlovian fear conditioning sessions (x-axis) is shown for Controls. Suppression ratios are reported as mean ± SEM. **(B)** The level of fear during pre-exposure and Pavlovian fear conditioning is shown for Early Adversity (EA) rats. All graph properties maintained from **(A)**. **(C)** The mean ± SEM suppression ratio to each cue (colors maintained from **A** and **B**) for Control (black outline) and EA rats (red outline) is shown for the last 8 sessions of discrimination. The asterisk indicates a significant difference between Control and EA rats (two-tailed *t*-test, *p* < 0.05). **(D)** Starting on the first pre-exposure session, we used a four-session sliding window analysis (n → *n* + 3) to calculate the difference in suppression ratios between EA and Control (Con) rats (y-axis, EA–Con). This difference was plotted for each cue [p(shock) = 1.00, red; p(shock) = 0.25, purple; and p(shock) = 0.00, blue]. A larger number indicates more fear to that cue by EA rats compared to Controls. The first (n) and last (*n* + 3) sessions of each four-session window are labeled on the x-axis beneath their corresponding data points. For every cue in each window, we tested the significance of the difference between EA and Con rats using a one-tailed, independent sample *t*-test. Asterisks indicate significance (one-tailed *t*-test, *p* < 0.05), and their colors denote the cue being compared. Blue # sign, *p* = 0.068. Purple # sign, *p* = 0.058.

In discrimination, rats were confronted with three cues: A1 always predicted shock, A2 predicted shock probabilistically, and A3 never predicted shock. Control rats rapidly suppressed nose poking to all three cues and maintained high levels of suppression to A1 throughout discrimination. Controls also successfully reduced suppression to both A2 and A3 such that by the eighth of sixteen discrimination sessions, they were showing intermediate but low suppression ratios to the uncertain A2 cue, and little or no suppression to the non-predictive A3 cue (Figure 2A). These levels were maintained until the end of discrimination. EA rats rapidly suppressed nose poking to all three cues and maintained high levels of suppression to A1 just as Controls. However, EA rats were slower to reduce suppression to cues A2 and A3. While EA rats did fully reduce suppression to A3 by the end of discrimination, they persisted in showing an elevated suppression ratio to the uncertain A2 cue throughout discrimination (Figure 2B).

In order to be certain any differences in body weight following early adversity did not drive our effects, the 85% body weight for each rat was used as a covariate in ANCOVA. In support of our description above, ANCOVA for suppression ratios [covariate: 85% body weight; within factors: session (18) and cue (A1 vs. A2 vs. A3); between factor: group (Control vs. EA)] found significant effects of body weight, group, cue, and the session × cue interaction (Fs > 1.5, ps < 0.05), but most critically a significant session × cue × group interaction [*F*_(34, 646)_ = 1.54, *p* < 0.05]. The significant interaction was driven by higher suppression ratios to the A2 cue by EA rats in the last half of the discrimination sessions (Figure 2C). Consistent with this interpretation, a contrast of the ANCOVA results isolating suppression ratios to the A2 cue by Control and EA rats over both halves (first and last eight sessions) of discrimination [factors: (Control: -1, EA: +1), (first half: -1, second half: +1), and (A1: -1, A3: -1, A2: +2)] was found to be significant [*F*_(1, 19)_ = 5.63, *p* < 0.05]. Significance was not found when contrasts examined differences in suppression ratios to the A1 or A3 cues (Fs < 2.0, ps > 0.2). Finally, consistent with both the ANCOVA result and planned comparisons, *post-hoc* tests of suppression ratios to the A2 cue over the final half of discrimination revealed significant differences between Control and EA rats [*t*_(20)_ = −2.27, *p* < 0.05].

To provide a finer temporal analysis of the differences between Control and EA rats over discrimination, we performed a sliding window analysis for each cue. There were no differences in suppression ratios to the A1 cue between Control and EA rats at any point during discrimination (Figure 2D–red line). EA rats were significantly slower to reduce suppression ratios to the A3 cue early in discrimination, but levels were statistically indistinguishable by the last four-session window of discrimination (Figure 2C–blue line). Most critically, a difference in suppression ratios to the uncertain A2 cue emerged later in EA rats, but was maintained for the duration of discrimination (Figure 2C–purple line).

### Extinction

We focused our extinction analyses on suppression ratio to the A1 cue because −APEs should most strongly contribute to extinction to this cue. EA rats also showed significantly higher terminal suppression ratios to the uncertain A2 cue, making comparisons in extinction inherently unfair. Because −APEs, should act entirely within a session, we first analyzed only the first session of extinction. One control rat failed to recall nose poke suppression to the A1 cue and was excluded from extinction analyses. Results were unambiguous, Control, and EA rats showed equivalent recall of nose poke suppression on the first trial. Regardless of group, rats showing the lowest levels of suppression to the uncertain cue during discrimination, showed the greatest reduction of suppression during the first extinction session.

**Figure 3:**
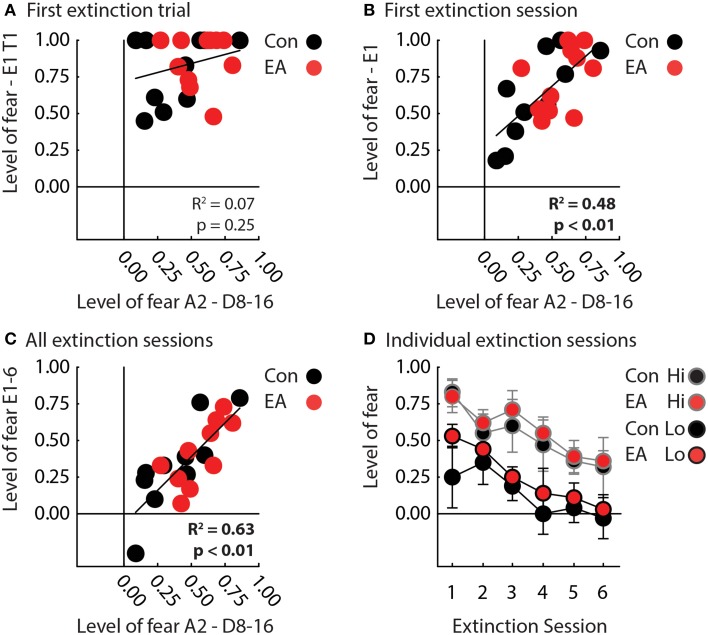
**Fear in extinction. (A)** A scatterplot compares mean nose poke suppression to the A2 cue during the final half of discrimination (x-axis) to mean nose poke suppression to the A1 cue on the first extinction trial for Control (black) and EA rats (red). There was zero correlation between the two measures across all rats (*R*^2^ = 0.07, *p* = 0.25). **(B)** A scatterplot compares mean nose poke suppression to the A2 cue over the final half of discrimination (x-axis) to mean nose poke suppression to A1 during the first extinction session for Control (black) and EA rats (red). There was a significant, positive correlation between the two measures across all rats (*R*^2^ = 0.48, *p* < 0.01). **(C)** A scatterplot compares mean nose poke suppression to A2 over the final half of discrimination (x-axis) to mean nose poke suppression to A1 over all extinction sessions for Control (black) and EA rats (red). There was a significant, positive correlation between the two measures across all rats (*R*^2^ = 0.63, *p* < 0.01). Note that one Control rat achieved a negative suppression ratio, indicating he increased, rather than suppressed, nose poking during the cue in extinction. **(D)** Mean nose poke suppression to A1 (y-axis) over the six sessions of extinction is plotted for Control (black fill) and EA rats (red fill) that showed Lo (black outline) or Hi (gray outline) fear to the uncertain A2 cue in the final half of discrimination (individual data taken from Figure 2C). “Lo” rats were the half of each group (Control, *n* = 5; EA, *n* = 5) showing the lowest levels of fear to the probabilistic cue in discrimination. “Hi” rats were the half of each group (Control, *n* = 5; EA, *n* = 5) showing the highest levels of fear. Rats were separated into “Lo” and “Hi” for illustrative purposes only. ANCOVA was performed with the level of fear to the probabilistic cue as a covariate.

In support, ANCOVA for suppression ratio to the A1 cue [covariate: mean suppression ratio A2 cue—discrimination sessions 8–16; within factor: trial (4); between factor: group (Control vs. EA)] revealed a significant effect of the covariate [*F*_(1, 18)_ = 15.50, *p* < 0.05] and a covariate × trial interaction [*F*_(3, 54)_ = 3.28, *p* < 0.05]. The interaction was the result of nose poke suppression recall on trial 1 being equivalent in all rats (*t*-test, *t* = 0.73, *p* = 0.47; Figure 3A), but greater extinction on subsequent trials by rats previously demonstrating low suppression ratios to the probabilistic cue during the last eight sessions of discrimination (Figure 3B). Interestingly, there was no effect of or interaction with group for this analysis (Fs < 2, ps > 0.2).

The same pattern was observed over all six extinction sessions. Regardless of group, rats that showed the lowest suppression ratios to the uncertain A2 cue during discrimination, showed the greatest reduction of nose poke suppression during extinction (Figures 3C,D). In support, ANCOVA for suppression ratio to the A1 cue [covariate: mean suppression ratio A2 cue—discrimination sessions 8–16; within factor: session (6); between factor: group (Control vs. EA)] found a significant effect of session [*F*_(5, 90)_ = 3.47, *p* < 0.05] and of covariate [*F*_(1, 18)_ = 28.97, *p* < 0.05]. No effect of or interaction with Group was found (Fs < 1, ps > 0.4). To visualize this statistical pattern, we separately plotted suppression ratios in extinction for Control and EA rats showing the highest (gray outlines) and lowest (black outlines) nose poke suppression to the probabilistic cue in discrimination (Figure 3D).

## Discussion

In the current study, we gave adolescent rats a battery of early adverse experiences and allowed them to mature without interruption. In adulthood, we assessed their ability to acquire and reduce fear to cues signaling different probabilities of aversive foot shock. Most challenging was a cue that predicted shock with uncertainty, requiring the use of −APEs to reduce fear. Normal rats initially acquired fear to all three cues, but showed appropriate levels of fear to each midway through discrimination: high fear to the fully-predictive cue, little or no fear to the non-predictive cue, and modest fear to the uncertain cue. Controls maintained these levels for the remainder of discrimination. The modest level of fear to the uncertain cue suggests Controls successfully used −APEs to reduce fear. Interestingly, EA rats were able to acquire fear, but were impaired in the reduction of fear to the uncertain cue and even the non-predictive cue. While EA rats eventually, showed little or no fear to the non-predictive cue, they continued to show elevated fear to the uncertain cue, even by the end of discrimination. This pattern is consistent with impaired use of −APEs to reduce fear. Early in discrimination, such a prediction error might be generated when no shock occurs on both non-predictive and uncertain trials. However, the use of −APEs would only persist for the uncertain cue throughout discrimination.

An alternative account of our findings is that early adversity *enhanced* the efficacy of +APEs. As a result, shock delivery on uncertain trials would be *more* aversive, permitting a stronger association between the uncertain cue and shock, resulting in higher levels of fear. This could also explain the slower fear extinction we observed in rats showing higher fear to the uncertain cue in extinction. In high fear rats, enhanced +APEs may have resulted in the formation of a stronger cue-shock association. Thus, extinction to the fully-predictive cue occurred more slowly because it started off with a stronger cue-shock association. However, if enhanced efficacy of +APEs was solely driving our effects, we would have expected EA rats to acquire fear more rapidly or perhaps demonstrate higher terminal levels of fear to the fully-predictive cue, which did not occur. It is also less straightforward how enhanced +APEs would impair reduction of fear to the non-predictive cue that is never followed by foot shock. Of course, these accounts are not mutually exclusive. Early adversity may both impair the use of −APEs and enhance the use of +APEs, both of which would work to increase fear in uncertainty.

Using the same behavioral design, we have previously found that fear to an uncertain cue in discrimination, a read-out of the ability to use −APEs to reduce fear, predicts extinction in normal rats (Berg et al., 2014). Somewhat surprisingly, early adversity had no impact on extinction independent of the demonstrated ability to use −APEs to reduce fear in uncertainty. This is not to say that Early Adversity has no effect on fear extinction. Indeed, the best individuals in extinction were Control rats showing the lowest fear to the probabilistic cue in discrimination (Figure 3B). Instead, these results suggest the effect of Early Adversity on fear extinction may be indirect; altering the neurobehavioral circuit underlying −APEs, which in turn impairs performance in extinction. Of course, the behavioral design we employed used far more cues and conditioning sessions than typical fear extinction studies (Corcoran and Maren, 2001; Milad and Quirk, 2002; Sotres-Bayon et al., 2007). In addition, the effect of Early Adversity on behavior may be stronger when closer to the event and decrease with time, making the effect on extinction comparatively weaker. Future studies comparing performance in our probabilistic procedure with separate experiments employing more traditional fear extinction procedures will provide more insight into this relatively new idea.

The early adverse experiences used here were chosen because previous studies have found them to be reliably stressful events (D'Angio et al., 1987; Melia et al., 1994; Vazdarjanova et al., 2001; Kelly et al., 2011). Importantly, none of these experiences involved the use of foot shock, which served as the aversive event during Pavlovian conditioning. Our findings of impaired fear reduction in EA rats cannot be described in terms of alterations in the processing of foot shock itself. Instead, early adversity has the ability to increase fear to uncertain cues predicting novel, aversive events. This is significant because some studies have found that increased risk for PTSD following trauma, specifically, assault/inter-personal violence, is only relevant if the early adverse experience is similar to the previously experienced trauma (Cougle et al., 2009). Although, it is impossible to directly compare an adverse-experience rodent study to human trauma case studies involving assault, the present data provide preclinical evidence that early adverse experiences can alter learning about, and fear to, aversive outcomes never previously experienced. This is more consistent with the idea that the specific nature of the early adverse experiences matters less than the number or severity of these events (Breslau et al., 1999; Xie et al., 2012).

Using the same probabilistic procedure, we have recently demonstrated a critical role for the dorsal raphe nucleus (DRN) in the reduction of fear through −APEs. Mirroring EA rats in the current study, rats with neurotoxic DRN lesions were specifically impaired in reducing fear to a probabilistic cue (Berg et al., 2014). Taken with the current findings, these results suggest that involvement of the DRN in generating or using −APEs to reduce fear may be specifically impaired by early life adversity. A role for the DRN as a learning signal for reducing fear is buoyed by its connectivity. The DRN projects to brain regions critical to fear learning such as the basolateral amygdala (Goosens and Maren, 2001; Gale et al., 2004; McDannald and Galarce, 2011), central amygdala (Wilensky et al., 2006; McDannald, 2010), and paraventricular thalamus (Do-Monte et al., 2015); as well as regions critical to fear extinction such as the infralimbic cortex (Milad and Quirk, 2002) and hippocampus (Corcoran et al., 2005). Recent studies implicate the ventrolateral periaqueductal gray (vlPAG) in the generation and use of +APEs (McNally and Cole, 2006; Cole and McNally, 2009; Johansen et al., 2010; Roy et al., 2014). Because, the DRN and vlPAG are strongly interconnected (Vertes, 1991; Ogawa et al., 2014), it is possible the DRN may serve to enhance or modulate the computation of vlPAG-produced +APEs. This would be consistent with the alternative hypothesis of enhanced +APE signaling brought on by early adversity. Future studies will specifically address the influence of early adversity on ±APE signaling in the DRN and vlPAG.

Here, we asked *if* Early Adversity impaired the ability to reduce fear to cue predicting shock probabilistically. The answer to this is unequivocally “yes.” Now that this has been demonstrated, the next logical question is *how* does this impairment come about? One hypothesis we propose is that Early Adversity impaired the ability to generate or utilize −APEs to reduce fear. However, other explanations are possible. Early adversity may generally impair sensory discrimination or probability detection. This could be tested by employing a procedure similar to that here, only using rewards instead of aversive foot shock. Early Adversity could also generally elevate anxiety, increasing fear to all cues present. It's possible this drove the results of this study, only observing an increase in fear to the 100% cue was not possible because fear levels were near ceiling. Along these lines, it would be informative to see if Early Adversity impaired various forms of safety learning (Christianson et al., 2011; Kazama et al., 2013; Sangha et al., 2013). Of course, other impairments are also possible. Critically, the current results provide a foundation for future studies specifying the neurobehavioral dysfunction brought about by Early Adversity.

Early Adversity undoubtedly results in many behavioral and neural changes that increase the risk for adult PTSD. The current results are significant because they demonstrate one specific change may be to impair the use of learning signals—negative, aversive prediction errors—to weaken fearful associations and ultimately reduce fear. Future studies identifying the neural origins of APEs, and their corruption by early adversity, will provide novel targets and more effective pharmacotherapies for PTSD and related anxiety disorders.

## Author contributions

MM conceived the work, MM/KW/AD acquired, analyzed, interpreted the work, MM/KW/AD drafted and critically revised the manuscript. MM/KW/AD approved the final manuscript. MM/KW/AD are accountable for all aspects of the work.

### Conflict of interest statement

The authors declare that the research was conducted in the absence of any commercial or financial relationships that could be construed as a potential conflict of interest.
